# [Retracted] Mechanism of cell death induced by silica nanoparticles in hepatocyte cells is by apoptosis

**DOI:** 10.3892/ijmm.2026.5730

**Published:** 2026-01-12

**Authors:** Ye Yang, Xinjing Du, Qiang Wang, Jianwei Liu, Enguo Zhang, Linlin Sai, Cheng Peng, Martin F. Lavin, Abrey Jie Yeo, Xu Yang, Hua Shao, Zhongjun Du

Int J Mol Med 44: 903-912, 2019; DOI: 10.3892/ijmm.2019.4265

Following the publication of this article, a concerned reader drew to the Editor's attention that the image showing silica nanoparticles in Fig. 1 on p. 906 had also been used to show the same data in another paper published by the same research group in *International Journal of Molecular Medicine*. Upon performing a separate investigation of the data in this paper in the Editorial Office, it also came to light that flow cytometric plots featured in Fig. 3 on p. 908 had originally been included in a paper featuring some of the same authors that had already been published in *International Journal of Nanomedicine*, and western blot data featured in Fig. 7 on p. 910 were originally included in another paper featuring some of the same authors in the journal *Stem Cell Research & Therapy*.

Given the apparent re-use of the abovementioned data in this article from previously published papers, the Editor of *International Journal of Molecular Medicine* has decided that this article should be retracted from the Journal. The authors were asked for an explanation to account for these concerns, but the Editorial Office did not receive a reply. The Editor apologizes to the readership for any inconvenience caused.

